# Neural network approaches, including use of topological data analysis, enhance classification of human induced pluripotent stem cell colonies by treatment condition

**DOI:** 10.1371/journal.pcbi.1012801

**Published:** 2025-07-11

**Authors:** Alexander Ruys de Perez, Paul E. Anderson, Elena S. Dimitrova, Melissa L. Kemp

**Affiliations:** 1 Mathematics Department, Bailey College of Science and Mathematics, California Polytechnic State University - San Luis Obispo, San Luis Obispo, California, United States of America; 2 School of Mathematics, Georgia Institute of Technology, Atlanta, Georgia, United States of America; 3 Department of Computer Science and Software Engineering, College of Engineering, California Polytechnic State University - San Luis Obispo, San Luis Obispo, California, United States of America; 4 Wallace H. Coulter Department of Biomedical Engineering, Georgia Institute of Technology and Emory University, Atlanta, Georgia, United States of America; University of North Texas, UNITED STATES OF AMERICA

## Abstract

Understanding how stem cells organize to form early tissue layers remains an important open question in developmental biology. Helpful in understanding this process are biomarkers or features that signal when a significant transition or decision occurs. We show such features from the spatial layout of the cells in a colony are sufficient to train neural networks to classify stem cell colonies according to differentiation protocol treatments each colony has received. We use topological data analysis to derive input information about the cells’ positions to a four-layer feedforward neural network. We find that despite the simplicity of this approach, such a network has performance similar to the traditional image classifier ResNet. We also find that network performance may reveal the time window during which differentiation occurs across multiple conditions.

## Author summary

Our understanding of how stem cells determine what specialized cells to differentiate into is still incomplete. One aspect of understanding this process involves identifying when key decisions about a cell’s fate occur. We explored whether by looking at the layout of the cells of the colony, we can infer knowledge regarding the eventual phenotypes the cells are differentiating towards. We train an algorithm to recognize cell type using spatial information by taking as input the number and size of holes that appear among the colony’s cells. We find this method succeeds in its classification, similar to an industry-grade image classifier.

## Introduction

Organoids and microphysiological systems generated from human induced pluripotent stem cells (hiPSCs) hold promise for developing in vitro assays that can be used for evaluating therapeutics, toxicological screening, and regenerative medicine. Furthermore, the way in which stem cells organize themselves into more specialized tissues under various culture conditions provides critical insight into morphogenesis, the dynamic formation of organ systems. During these processes, guidance to individual cells comes through positional cues [1] and interactions with neighbors [2]. Thus, while morphogenesis is driven by soluble morphogen signals, the process itself can be readily observed through spatial information in the form of cell migration, clustering, and changes in densities. We ask whether this spatial information can be interpreted in a way that allows us to determine when the differentiation process is precisely occurring, and what differentiation is taking place. Ultimately, the prediction of eventual lineage specification by the simple non-invasive metrics derived from multicellular organization is desired for reliability and real-time quality control of organoids in an industrial manufacturing setting.

In responding to the challenge of tissue identification from spatial data, we design a neural network which takes as input the positional data of a colony of cells, and guesses the cell fate of the colony. Applying deep learning to these image classification problems has yielded success. Image classifying neural networks have been able to identify cell morphology [3], and have found applications in cell sorting [4] and cytopathology [5]. Especially relevant to our interest is the work of [6], which predicted the differentiation of individual primary murine hematopoietic stem and progenitor cells (HSPCs) into one of two cell fates: the granulocytic/monocytic (GM) or the megakaryocitic/erythroid (MegE). In this case deep learning could accurately predict the lineage three generations before the cells began emitting the identifying markers.

We venture from the previous work by using deep learning to make conclusions about a mass of many related cells, rather than a single individual cell. Morphology has provided information about differentiation of pluripotent stem cell aggregates. In [7], classification of embryoid bodies by morphology (cystic, bright cavity, or dark cavity) predicted which early germ layers would emerge. This result introduces the potential of a computer to make further predictions based on details not evident to human observation. While a natural approach to this machine learning problem would be to design an image classifier, we also include a network that uses topological data analysis (TDA). We hypothesize that the *persistent homology* of the colony, which effectively catalogues the gaps and holes that form between the cells, will distill the critical features that would be lost in the raw image. Persistent homology has proven to be insightful in other biological contexts. Indeed, [8] found that a support vector machine could use persistent homology, when refined into a persistence landscape function, to distinguish between open and closed conformations of the maltose-binding protein. On the level of cellular interactions, [9] found that 1-dimensional homology could serve as an effective classifier for motility phases in epithelial cells.

## Results

### Both the topological data-using network and the standard image classifier succeed in classifying images

In order to investigate the potential utility of TDA in detecting changes in stem cell aggregates associated with culturing protocols, we performed a comparative analysis between a standard image classifying neural network (ResNet) and a simple feedforward neural network (TDANet) which used our topologically-derived feature set. Frames from a previously published study [10] of time-lapsed microscopy of iPSC aggregates undergoing differentiation from 5 experimental conditions over 48 hours were analyzed for 0th order and 1st order homology; this information was used to train TDANet as described in Materials & Methods. Alternatively, the images were directly fed into ResNet. A model was thus trained, for some particular timepoint, on the images of the 78 colonies (in the case of ResNet) at that timepoint or on each homological data summary of the 77 colonies (in the case of TDANet) from that timepoint (one colony was missing its homological data). See the Processing Coordinate Data subsection of Materials & Methods for a description of the homological data summary.

**Fig 1:**
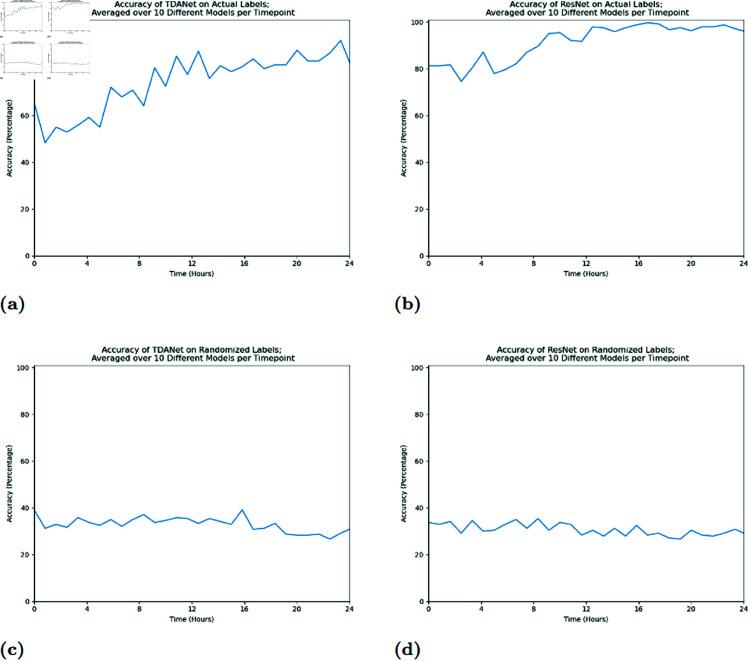
Average accuracy of TDANet and ResNet models trained at a fixed time on time series hiPSC data from [10]. For each timepoint, ten different neural network models were trained on data from the hiPSCs at that timepoint. Data from each timepoint was divided such that 70% were used for training and 30% for validation with the divisions randomized for each instance. The above graphs show the average accuracy of predictions using TDANet models (A and C), as well as ResNet models (B and D). Results are shown for both the case when a model was trained on the correct labelings of the colonies (A and B) and for the case when trained on a randomized re-labeling of the colonies (C and D). The TDANet models used a combination of 1-dimensional and 0-dimensional homology data.

We found that both neural networks were successful in classifying the colonies. As expected, the networks tended to perform more poorly at earlier timepoints, when the colonies were mostly pluripotent and thus hadn’t organized their distinguishing features. At later timepoints classification accuracy increased to about 80% accuracy in the case of TDANet and over 90% in the case of ResNet. Furthermore, low validation accuracy from a parallel training session where the colonies were randomly assigned a treatment label (see the Training and Randomized Labels subsection of Materials & Methods) showed the networks were dependent on the underlying biological information. As Fig 1C and 1D show, on the randomized labels neither TDANet and ResNet could achieve an accuracy little better than randomly choosing one out of five. We conclude that the networks cannot find similarities in arbitrary groupings of colonies. This suggests that the accuracy shown when trained on the colonies’ correct labels comes from information related to the biology of the cells, and not incidental patterns specific to the data.

While ResNet tends to have better results than TDANet, both networks exhibit the same behavior. In particular, both networks’ accuracies are characterized by two plateaus. Between timepoints *t*001 and *t*100 (from the beginning of recording to approximately 8.3 hours after), the accuracy hovers around a score of ~60% for TDANet and ~80% for ResNet, before jumping to their respective optimized rates during the interval of *t*100 to *t*150 (approximately 12.5 hours into imaging). This interval is of interest since it suggests that both networks are sensitive to cell differentiation in this experimental window. That is, the timepoints during which accuracy increases from the lower plateau to the higher plateau might be the window during which the colonies differentiate from their pluripotent and less distinguishable beginnings into their final tissue fates. Past work has shown that information about cell fates appears well before differentiation occurs. For example, reading gene expression levels during pluripotency could predict the percentage of cells that differentiated into cardiomyocytes [11] and hepatocytes [12]. Our work suggests that these clues can be seen in the morphology as well.

**Fig 2:**
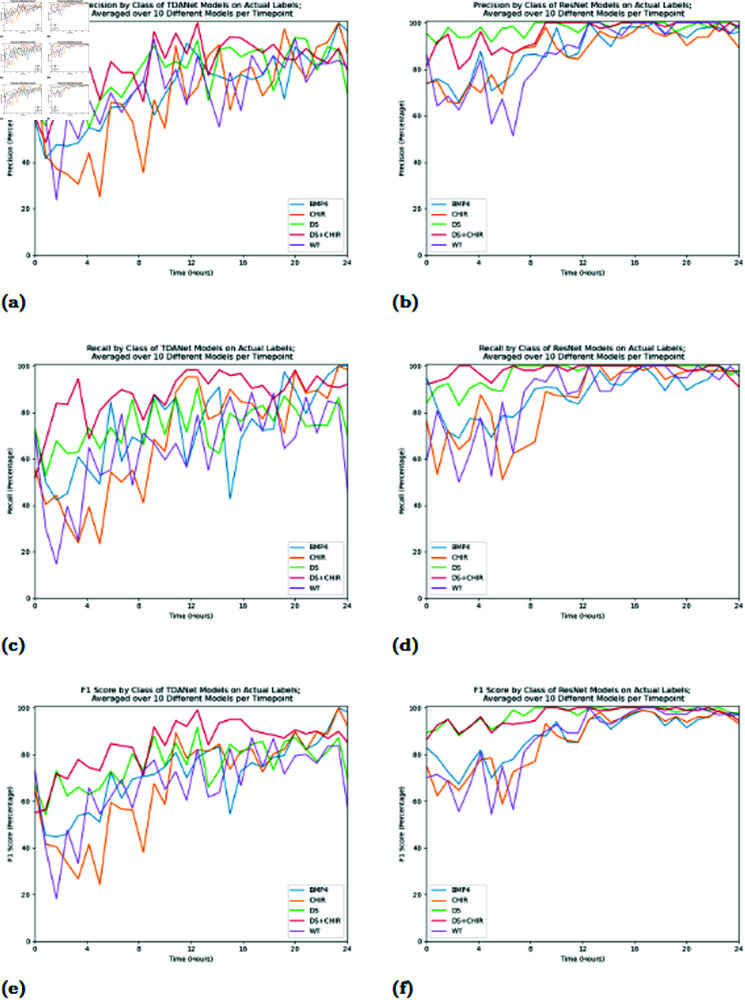
Average precision (A and B), recall (C and D), and F1 scores (E and F) by class of TDANet and ResNet models on the validation data from the training exercise that generated the results from Fig 1. The above graphs show these metrics for TDANet models (A, C, and E), as well as ResNet models (B, D, and F). Results are shown only for the case when a model was trained on the correct labelings of the colonies.

As Fig 2 shows, the performance of the models on an individual class tended to follow the pattern of the performance of the colonies in general. The F1 scores in most cases were low at earlier times, increased between hours 8 and 12, and then plateaued at the later timepoints. The precision, recall, and F1 score of a particular class are defined as follows:


$$\text{precision}=\frac{\text{\# of true positives}}{\text{\# of true positives }+\text{\# of true negatives}},$$

$$\text{recall}=\frac{\text{\# of true positives}}{\text{\# of true positives }+\text{\# of false negatives}},\text{ and}$$

$$\text{F1}=\frac{2*\text{precision}*\text{recall}}{\text{precision}+\text{recall}}.$$

**Fig 3:**
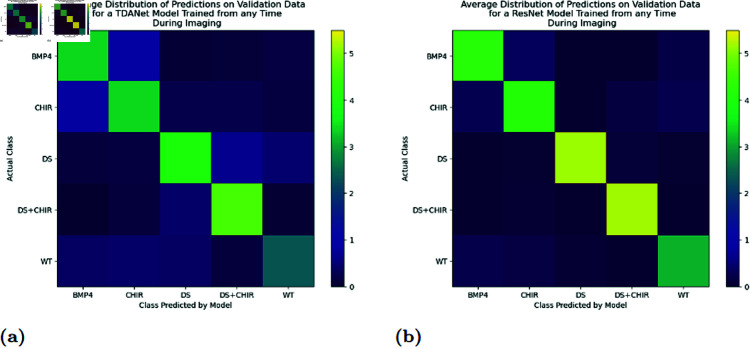
Confusion matrices for TDANet (A) and ResNet (B) when tested on the validation data set. The models used here were those trained on the actual labels in the same training exercise that generated the results in Figs 1 and 2. The value in each square is the average number of colonies of the class type on the vertical axis and predicted by the model to be of the class type on the horizontal axis.

The major exceptions were the DS and DS+CHIR classes when analyzed by ResNet, which at the earlier timepoints held a remarkable lead over the other three classes. This behavior is supported by the confusion matrices in Fig 3. The matrices show that there were two main errors: both models confusing BMP4 with CHIR, and TDANet confusing DS and DS+CHIR. ResNet did not appear to have the difficulties in distinguishing DS from DS+CHIR that TDANet had, thus accounting for the very high scores for these classes.

**Fig 4:**
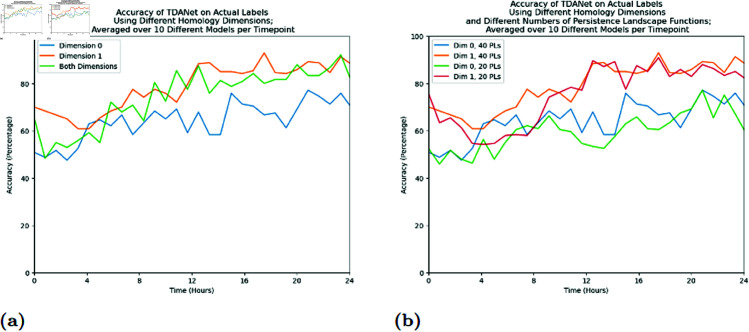
(A) Accuracy of TDANet models when trained on just the dimension 0 or just the dimension 1 homology data, compared to the models trained on a combination of data from both dimensions. (B) Comparison of TDANet models trained on using the first 40 persistence landscape functions as opposed to just the first 20 persistence landscape functions. In B every model is trained on data from just one of the homology dimensions.

An unexpected result of TDANet was the impact of the dimension of the homology data. We had expected TDANet to perform best when given an input of both 1-dimensional and 0-dimensional homology. However, the accuracy of TDANet when trained on this comprehensive data does not significantly exceed its accuracy when given homology data from just one of the dimensions (see Fig 4A). In particular, TDANet trained on both 0-dimensional and 1-dimensional homology data does not outperform TDANet trained on just the 1-dimensional data. While the model using both dimensions only takes the first twenty persistence landscape functions for each dimension instead of the first forty, this does not appear to negatively impact its performance. Indeed, we created a version of TDANet that would just take the first twenty persistence landscape functions of a single homology dimension. We found that the performance of this model approximated the version that used forty persistence landscape functions (see Fig 4B), suggesting that the critical knowledge for classification resides in the initial landscape functions. As an alternate explanation, we believe that much of the same information about the colony is encoded in both dimensions, so including the persistence landscape functions of more than one is redundant.

### Attention analysis suggests topological data can improve use of biological features for ResNet classification

We used Smooth Grad-CAM++ [23] to create class activation maps showing ResNet’s analysis of the colony images. Class activation maps (CAMs) are a useful tool that allow further insight into the decisions made by an image classifier like ResNet. The CAM acts as a heatmap laid over the original image, showing the attention paid by the network to the different parts of the image. The value of the CAM at a specific location is dimensionless; it is a normalized score that shows the importance to the classifier of that particular point relative to other locations. Regions that have high values are those that make a larger contribution to the net’s classification decision [13]. In evaluating the CAMs, one can thus determine what parts of the colony are the most impactful in distinguishing it from colonies of different treatment types. Furthermore, the CAM can also show whether the network is making the categorization using the “correct” data. That is, if high attention is paid to regions with cells, then this suggests the network is making its decisions based on the underlying biological phenomena in the image. On the other hand, if the heatmap tends to focus on areas that have no cells, this implies the network is using details specific to the image, like the location of the colony in the photo.

**Fig 5:**
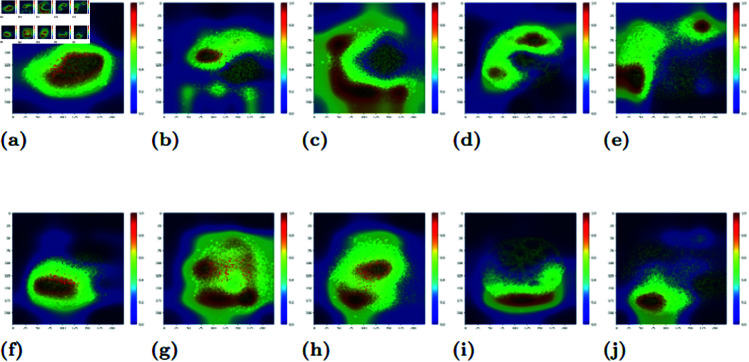
Examples of class activation maps of the five different treatment protocols. Top row (A,B,C,D,E) is the image created by the network trained on the actual labels, while the bottom row (F,G,H,I,J) consists of the same images trained on randomized labels. The columns are arranged by protocol treatment. In order they are WT (A and F), BMP4 (B and G), CHIR (C and H), DS (D and I), and DS+CHIR (E and J).

In general, the CAMs reveal that ResNet generally focuses on the region of the image that contains the cells. This can be ascertained via a side-by-side comparison of ResNet models with different training protocols, some examples of which can be seen in Fig 5. More CAMs, including movies showing the evolution of CAMs over time for a colony, can be found at https://doi.org/10.5281/zenodo.15307031. The movies show that in contrast to the ResNet models that were trained on randomized labels, the models trained on the correct labels are much more consistent with respect to their distribution of attention.

Each of the different protocol treatments has its own patterning in the class activation map. In the case of the WT and DS-treated colonies (see the Data subsection of Materials & Methods for a description of the different treatments), the high-value region of the class activation map is centered on the interior of the colony. The difference between these two types lies in the spread of attention across the colony. For the WT colonies, almost the entire aggregate of cells has a relatively high activation value. However, in the case of the DS colonies, there are large areas of the interior that have an activation value close to 0.

In the case of the BMP4 and CHIR colonies, the CAMs tend to place their attention on the border region of the colony, with less significance given to the interior. This pattern can still be evident of the net’s receptivity to the colony structure: Both the BMP4 and CHIR treated colonies are characterized by a “fringe”, where the cells on the boundary are less densely distributed than the cells in the interior. We conclude that the CAM’s attention to the border demonstrates awareness and use of this feature in the classification process.

For the DS+CHIR colonies, attention tends to focus on the corners of the image. In other colonies these corner locations would have little to no cells, but due to the size of the DS+CHIR colonies they are populated. Thus we conclude that the network is characterizing the DS+CHIR colonies using its larger size, as only this treatment type would show a dense cell population in these areas.

A particularly striking conclusion about the CAMs for the DS treated colonies is that the selected areas the network does pay significant attention to are not necessarily the areas characterized by the “rosettes”. These low density holes that appear in the colony are eye-catching to a human observer and are a distinguishing feature for this type of treatment, as no other colony type shows this motif. It is a surprise then, that the network does not give much importance to these formations. However, in this shortfall we can see an opportunity for topological data analysis. The rosettes are a feature that would stand out in a barcode. The disregard paid them by ResNet shows that there is some relevant information in the topology that is not used by an image classifier.

In summary, the class activation maps reveal that ResNet uses information about the colonies for its classification of the images, and is able to distinguish colonies based on unique biological features. However, as shown in the maps of the DS+CHIR colonies, ResNet can obfuscate the biological data with circumstantial details. Also, as evidenced by the DS colonies, not all relevant details are fully picked up by ResNet. Thus, it appears as though ResNet could benefit from a more directed focus using topological information.

### Networks show similar performance for time-separated training and testing datasets

Another method to evaluate the networks’ robustness involves testing a model on data from a timepoint different from the timepoint on which the model was trained. Here, we train a model on colony data from *T*, then for a different time S≠T, we have the *T*-trained model classify the data from *S*.

The point of this exercise is to see how well insights made by the networks about the colonies extrapolate over time. We expect that the model trained on data from timepoint *T* will perform better on data from timepoints that are close to *T* compared to timepoints that are further away. We would like to see how quickly this performance decays; that is, how far away a timepoint from *T* can a model trained on *T* sustain high accuracy.

**Fig 6:**
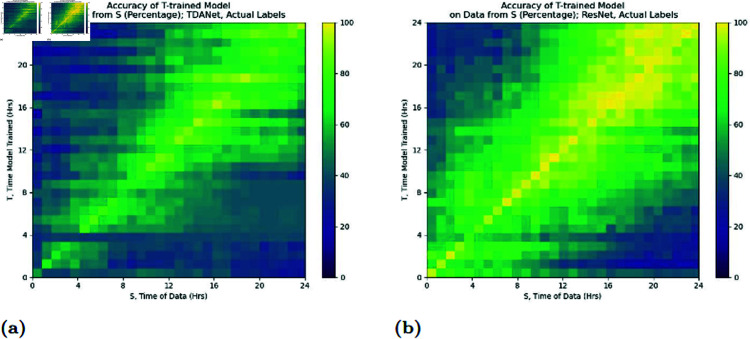
Accuracy of a neural network trained at timepoint *T* and tested on data from a different timepoint *S.* Training and testing were both done on the actual labels. Results are for both TDANet (A) and ResNet (B).

As Fig 6 shows, we do see a trend of models performing better on data from timepoints close to the time when they were trained. This nearby performance effect tends to remain stronger for models trained at later timepoints.

**Fig 7:**
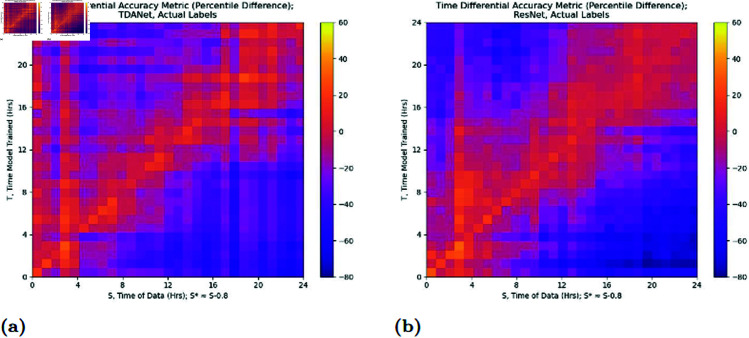
Time differential accuracy metric ⟨T,S⟩ for distinct timepoints *S* and *T.* A neural network is trained on data from *T*, along with another network trained on data from ${\text{S}}^{*}$, a time close to *S*. The resulting metric is the difference in accuracy between the *T*-trained network and the ${\text{S}}^{*}$-trained network on data from *S*. Results are for both TDANet (A) and ResNet (B) trained on the actual labels.

However, an issue with Fig 6 is that it masks the decay with its overall performance. It is possible that some models maintain robust performance with little decay in accuracy that is unseen because they have a lower starting accuracy. To address this we introduce an accuracy metric which we call the *time differential accuracy metric* ⟨T,S⟩ for two timepoints *T* and *S*. We define it as


$$\langle T,S\rangle \;={\text{Acc}}_{T}(S)-{\text{Acc}}_{S^{*}}(S),$$

where ${\text{Acc}}_{A}(B)$ is the accuracy of a network trained on data from time *A* when tested on data from time *B*, and ${\text{S}}^{*}$ is a time close to *S* (in our case 50 minutes earlier). The goal of this metric is to show the relative change in accuracy of the neural network model, as this better represents how much accuracy a network can preserve. We choose ${\text{S}}^{*}$ instead of *S* since using an *S*-trained model would suffer from one of two issues: either the testing dataset would include training data it had already seen, or with colony data split into disjoint training and testing sets it would thus be tested on a smaller dataset than the *T*-trained model. The results of the analysis using this metric are in Fig 7.

There are two observations to be made from the analysis concerning the rate at which model accuracy decays the further the testing time is from the training time. First, for both ResNet and TDANet, the decay rate is slower when the model is trained on data from later timepoints (the high values for the metric at early values of *S* are likely due to the poorer starting accuracy of the ${\text{S}}^{*}$-trained model, as evidenced by the fact that values remain high for all *T*). Second, the decay rate is not appreciably different between ResNet and TDANet. The first result is expected for reasons similar to why we would expect higher accuracy at later timepoints: in this period the colonies at both *T* and *S* have differentiated, and so distinguishing features of treatments would be recognizable in both datasets. The second result is more ambiguous, though it seems to suggest that ResNet and TDANet have equal capability in transferring their insights across time. Perhaps this effect is due to limitations in similarity to colony features at different timepoints.

## Discussion

We investigate the potential for neural networks to accurately classify stem cell fates using morphological data. To do so, we take pluripotent stem cell colonies given one of five differentiation protocol treatments, and ask a neural network to guess the correct protocol. We compare two different network models, each with its own type of information used as input. One is ResNet, a traditional image classifier that uses a photo of the colony as input. The other is TDANet, a simple 4 layer feedforward network that uses topological information in the form of persistent homology created from approximations of the cells’ locations.

Results show that TDANet can classify stem cell colonies with a performance far better than random chance. Network performance is in keeping with assumptions about classification difficulty due to colony differentiation, with networks trained on data from later timepoints tending to have higher accuracy rates. Furthermore, accuracy results reveal two time periods during which accuracy is stable, with a transition occurring roughly 8 to 10 hours after starting data collection. This transition, which can also be observed in the ResNet models, could be the window during which the colonies undergo differentiation, with the earlier time period being when the cells were mostly in their undifferentiated state, and the post-transition period when the colony has achieved its final state.

What is significant about the transition period is that it covers timepoints where many of the colonies’ images are still indistinguishable to the human eye. If indeed this transition is the period of differentiation, then this shows computer vision’s ability to detect structural differences in tissues that is not apparent to humans. Unfortunately, we cannot verify the moment of differentiation from colonies of this particular dataset. However, a straightforward follow-up experiment where differentiation status can be tracked and recorded could ascertain this, and provide a crucial insight.

In parallel with the conclusion about the transition period, however, one might also wonder why the networks can perform decently at the earlier, less differentiated states. That accuracy is well above the 20% success rate of making a random guess suggests the possibility of the networks overtraining. However, the imaging did not begin immediately at the beginning of the protocols but rather after a waiting period of at least 24 hours. Thus, some amount of differentiation would have occurred even early in the imaging process. One can observe from the sample images in “Data” of Materials & Methods that the colonies at early timepoints still show distinguishing motifs.

When compared to the results of ResNet, our TDANet clearly had lower accuracy. However, there are a couple of caveats to simply dismissing TDANet in favor of an image classifier. For one, as our analysis on the class activation maps alludes to, each network has its own set of insights used in the decision making process to which the other does not have access. Furthermore, given the difference in sophistication between the two different neural nets used, it would be inaccurate to conclude that the visual information is superior to the topological. It seems more likely that the performance gap is due to network architecture, and not data input. Indeed, we found that training the ResNet models took roughly ten times as long as training the TDANet models (see “Hardware and Network Training Specifications” of Materials & Methods). This leaves open a couple of doors to future work. For one, the performance of the topological method could be significantly improved if we used a more complex network. We ask whether TDANet can be modified or refined into a network that makes use of the persistent homology in a superior manner. Alternatively, topological data could be incorporated into an image classifier. In this conception the image classifier would be told to focus on those parts of the image that have significant homological elements, either through restricting the neural network to those regions or giving them greater weight during decision making.

## Materials and methods

### Persistent homology

**Fig 8:**
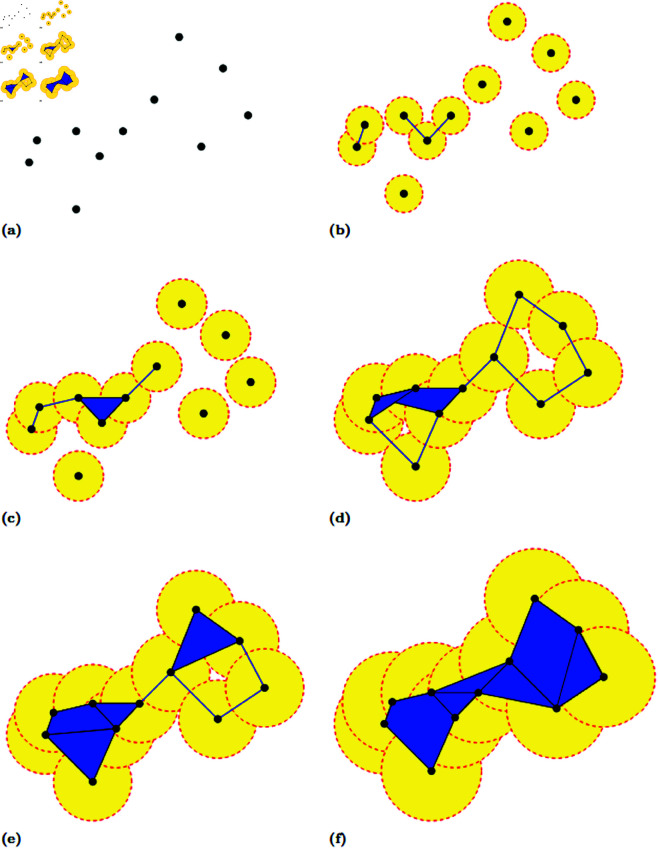
Stages of the growth of the Rips complex for a set of points *X* for increasing values of the radius of the circles used in computing the complex.

The mathematical tool we use in preparing the colony data is persistent homology. Here, we will present a simplified version for analysis of 2-dimensional data. Those wishing to learn more about the generalized approach should see Edelsbrunner *et al*. [14] Informally, the job of persistent homology is to provide quantitative data for properties of data sets that are usually described qualitatively. As an example, we begin with the point cloud *X* shown in Fig 8A. At a glance, the data takes the shape of what resembles a barbell, with two separate rings of points joined together. But how do we rigorously describe this? How can we make a case that the point at the very bottom of the figure is “close enough” to complete the lower left circle? How do we make the case that smaller rings, like a triangle made up of three of the points, are not as significant?

We draw a circle of radius *r*, where *r* is a parameter value, around each of the points. We start with a value for *r* so small that none of the circles intersect, and then observe the intersections of the circles as *r* increases.

When *r* becomes large enough such that two circles intersect, we place a line segment between the points for which those circles are the center. In this way we are keeping track of the total number of connected components. In this example, we begin with 11 different components, the points themselves. As points are joined by line segments, the number of components shrinks as they merge together. In Fig 8B, for example, the radius *r* of the circles is large enough that we place down three line segments.

In addition to placing line segments as *r* increases, we also place down polygons. Whenever we have a set of three or more points such that the pairwise intersection of any two of their circles is nonempty, we set down a polygon whose vertices are precisely those points. We can observe an example of this in Fig 8C. For the three points that were joined together in Fig 8C, their circles have now grown large enough that the intersection of any two is nonempty. We thus “fill in” the convex area between the three points to create a triangle. We continue to keep track of the components, noting that at this stage there are 6 components. With the addition of polygons, we are building what is called a *simplicial complex*. We call the simplicial complex constructed in this manner a *Rips complex*.

As we monitor the number of components, we also take note of instances where “holes” appear in our complex. One can see two examples of this in Fig 8D. By this point, the radii have grown large enough that all the points have been connected into one large component. However, the radii are not large enough that we have filled in all the convex areas between points with polygons. As such, the Rips complex has formed two loops that encircle regions of the plane not yet added to the complex. We keep track of the *persistence* of each of these holes through two values of *r*: the value at which it forms and the value at which it is filled in. As one can observe in Fig 8E, there is a value of *r* at which the bottom left has now been filled in, while the top right hole persists, albeit at a smaller size. It is not until a larger value of *r*, shown in Fig 8F do we have that this hole vanishes. At this point our Rips complex is a single connected component without any holes, and will remain as such as *r* continues on to infinity.

**Fig 9:**
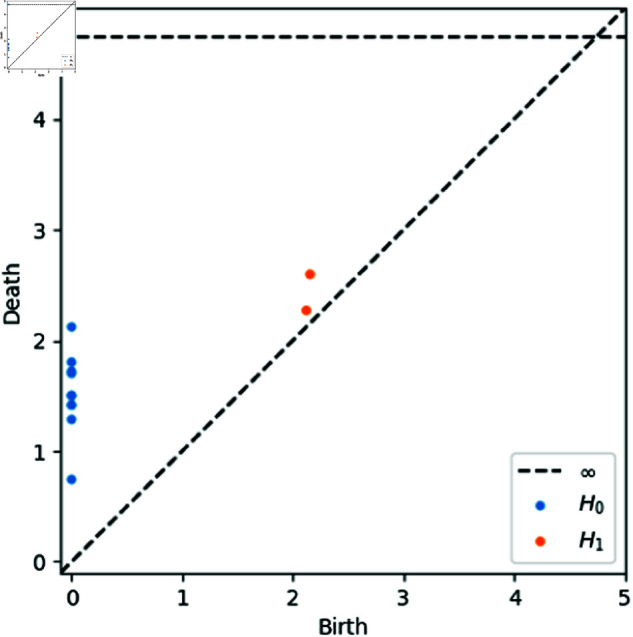
The persistence diagram for the point cloud *X* shown in Fig 8, computed using software from [15]. The blue points represent the connected components (0-dimensional homology elements, or *H*_0_) of the Rips complex. The orange points represent the holes (1-dimensional homology elements, or *H*_1_). The horizontal axis, labeled “Birth”, denotes the value of *r* at which the element appears (note this is why all the connected components are at *r* = 0, as they existed in the form of individual points at the very beginning). The vertical axis, labeled “Death”, denotes the value at which the element disappears. In the case of the components, this is the value at which it connects to another component. For the holes, this is the value at which the hole is “filled in”. The blue point on the dotted line labeled ∞ is the one large component into which all the components are eventually joined.

We summarize our analysis in a *barcode*, a collection of intervals with each interval representing the lifespan of a particular homology element (a connected component or a hole). The connected components are the *0-dimensional* elements and the holes are the *1-dimensional* elements. The interval’s endpoints are the “birth” and “death” of the corresponding element. These are respectively the value of *r* at which the element comes into being (*r* = 0 for the components; the *r* that connects the loop for the holes) and the value of *r* at which the element ceases to exist (the *r* at which the component merged with another or the hole is completely filled in). The barcode can be represented graphically as a *persistence diagram* [14], as shown in Fig 9.

With the barcode, we now have concrete metrics about the density and clustering of the data set and can thus elucidate its shape more clearly. Returning to the beginning of our example, we can see that we are justified in our characterization of *X* as two rings joined together. The barcode has precisely two 1-dimensional homology elements; the two rings we originally saw at first glance.

The approach we have detailed in the example is the one we take with our colony data. Like the data set *X* of the example, our raw data consists of points in the coordinate plane. Each data set consists of a single colony at a particular timepoint, and each point represents an approximation of the location of a cell nucleus. We construct a Rips complex and record the values of *r* at which the components and holes of the complex appear and disappear during its construction. We use the program Ripser for the computation of the complexes and the resulting barcodes [15].

### Data

We used data on 78 human induced pluripotent stem cell (hiPSC) colonies from the work of [10] as provided by the authors. Each of these colonies was either left as wild type (WT) or treated with one of four morphogen combinations: BMP4, dual SMAD inhibition (DS), CHIR, or combined dual SMAD inhibition and CHIR (DS+CHIR). Each of these morphogens affects differentiation during gastrulation. CHIR is an activator of the WNT pathway [16]. Suppression of BMP4 has been shown to lead to the failure of the mesoderm to develop [17], and SMAD features in the Nodal pathway, which promotes mesoderm formation [18,19]. The number of colonies per classification was WT: 12, BMP4: 16, CHIR: 16, DS: 17, DS+CHIR: 17. There was a single BMP4 colony for which we had the images but not the coordinate data. Thus this colony was used only in training ResNet.

**Fig 10:**
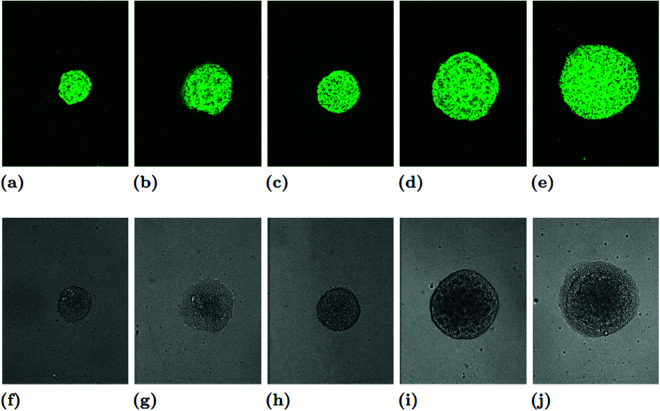
Examples of a colony with one of the 5 treatment types, when 50 minutes into imaging. Shown are both the images with green fluorescent protein labels (GFP) (A-E) and phase contrast images (F-J). The protocol for each colony is WT (A, F); BMP4 (B, G); CHIR (C, H); DS (D, I); DS+CHIR (E, J).

**Fig 11:**
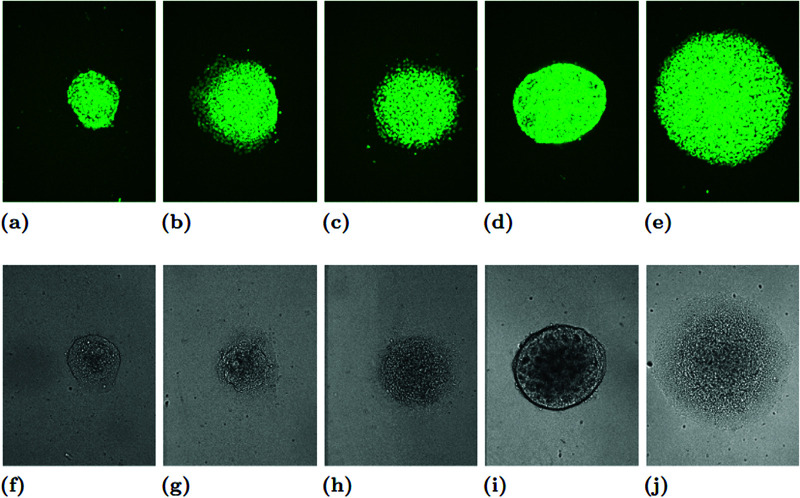
GFP and phase contrast images of the colonies from Fig 10, now taken when 11 hours and 40 minutes into imaging. The colony images are in the same order as in Fig 10: WT (A, F); BMP4 (B, G); CHIR (C, H); DS (D, I); DS+CHIR (E, J).

**Fig 12:**
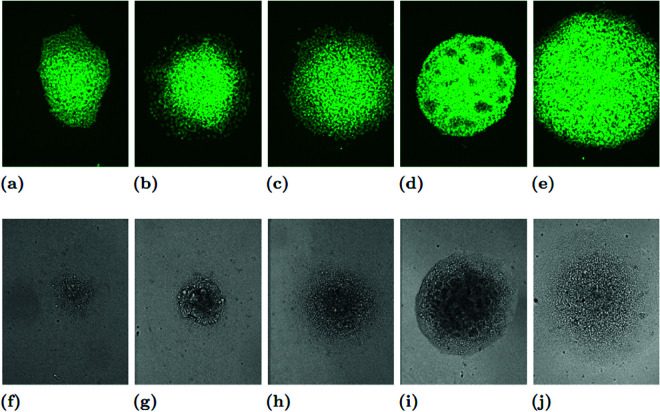
GFP and phase contrast images of the colonies from Fig 10, now taken when 22 hours and 30 minutes into imaging. The colony images are in the same order as in Fig 10: WT (A, F); BMP4 (B, G); CHIR (C, H); DS (D, I); DS+CHIR (E, J).

Every colony was imaged over a 24-hour period. Images of each colony were acquired every 5 minutes, for a total of 288 individual frames. The images, which were 834×1094 pixel photos of a 76.50×1002.83 micron space, were then analyzed for cell detection and segmentation by the authors to create a list of coordinates approximating the locations of each of the cells, which tended to number between 500 and 2000 per colony. Thus, each colony has 288 sets of coordinate data derived from 288 images, yielding 78 time series. See Figs 10, 11, and 12 for images of the colonies taken at the beginning, middle, and end of the imaging period, respectively. Images of all the colonies at these three specific times can be found in the links below (note that we used only the images with the green fluorescent protein (GFP) labels for training):

Early, GFP: https://doi.org/10.5281/zenodo.15306992
Early, Phase Contrast: https://doi.org/10.5281/zenodo.15306976
Middle, GFP: https://doi.org/10.5281/zenodo.15307001
Middle, Phase Contrast: https://doi.org/10.5281/zenodo.15306994
Late, GFP: https://doi.org/10.5281/zenodo.15307018
Late, Phase Contrast: https://doi.org/10.5281/zenodo.15307009

For each protocol treatment, the colonies were created by first aggregating the cells over a 24-hour period, after which the aggregates were plated down in culture wells and developed into colonies. In the case of the BMP4 and CHIR treatments, there was a 24-hour wait period, after which the ligands were applied for another 24 hours before the final 24-hour imaging process. In the case of the DS treatment, the treating of the cells occurred at the beginning of the aggregation period, and continued throughout the consecutive 24-hour periods of aggregation, post-seeding colony formation, and imaging. For the DS+CHIR treatment, CHIR was applied 48 hours before aggregation, and was maintained through the 48 hour pre-aggregation period, the 24 hour aggregation period, the 24 post-seeding period, and the 24 hour imaging period [10].

### Processing coordinate data

We used Ripser [15] to create a barcode from the coordinate data of a colony at a particular timepoint.

However, using a barcode as input to our feedforward neural network raises the difficulty of fitting a data structure of varying length to a fixed input size parameter. We wanted to avoid imposing an arbitrary cutoff on the number of intervals from a barcode given to a network. Thus, we implemented a new input format using the *persistence landscape*.

We define the *persistence landscape* [20] as follows. For an interval (*b*,*d*) in the barcode, define the function $f_{\left(b,d\right)}:[0,\infty )\to [0,\infty )$ to be


$$f_{\left(b,d\right)}(t):=\left\{ \begin{array}{cc} 0, & t\leq b\text{ or }d\leq t\\ t-b, & b<t\leq \frac{b+d}{2}\\ d-t, & \frac{b+d}{2}<t\leq d \end{array}\right.$$

The persistence landscape is then a family of functions $\{\lambda_{k}{\}}_{k=1}^{\infty }$ where the *k*th persistence landscape function is given by


$$\lambda_{k}(t):=\text{kmax}\{f_{\left(b,d\right)}(t)\}$$

over all persistence elements (*b*,*d*) in the barcode. Here, *kmax* means the *k*th largest element in the set.

Informally, the persistence landscapes are used here to find the long-lasting features, but in the context of comparing with features that occur for the same values of *r*. For example, say a barcode has an interval $\left[r_{0},r_{1}\right)$. Suppose that this interval, while shorter than many other elements of the barcode, is the longest such interval among intervals that extend into the region between *r*_0_ and *r*_1_. Then this interval would feature prominently in the persistence landscapes, as while it is not a large interval, it is a large interval in its locality.

**Fig 13:**
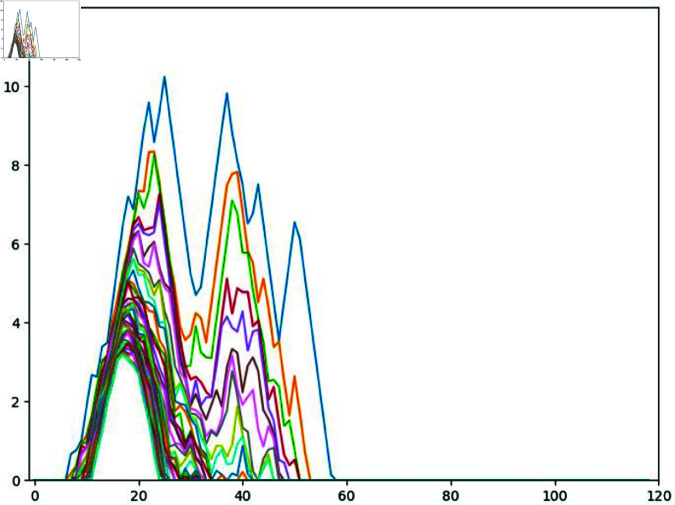
A graph of the first forty persistence landscape functions of the persistent homology of a stem cell colony.

We selected as sample points for the filtration parameter *r* the values 1,2,…,40 and the first forty persistence landscape functions $\lambda_{1},\lambda_{2},\ldots ,\lambda_{40}$. We decided on the number 40 in both cases based on examination of the persistence landscape functions of the colonies at various timepoints, which found little information for parameter values greater than 40 or for $\lambda_{i}$ when *i* > 40 (see Fig 13 as an example). The input to the neural network is thus a 40×40 matrix whose (*i*,*j*)th entry is $\lambda_{i}(j)$.

### TDA-using neural network

**Fig 14:**
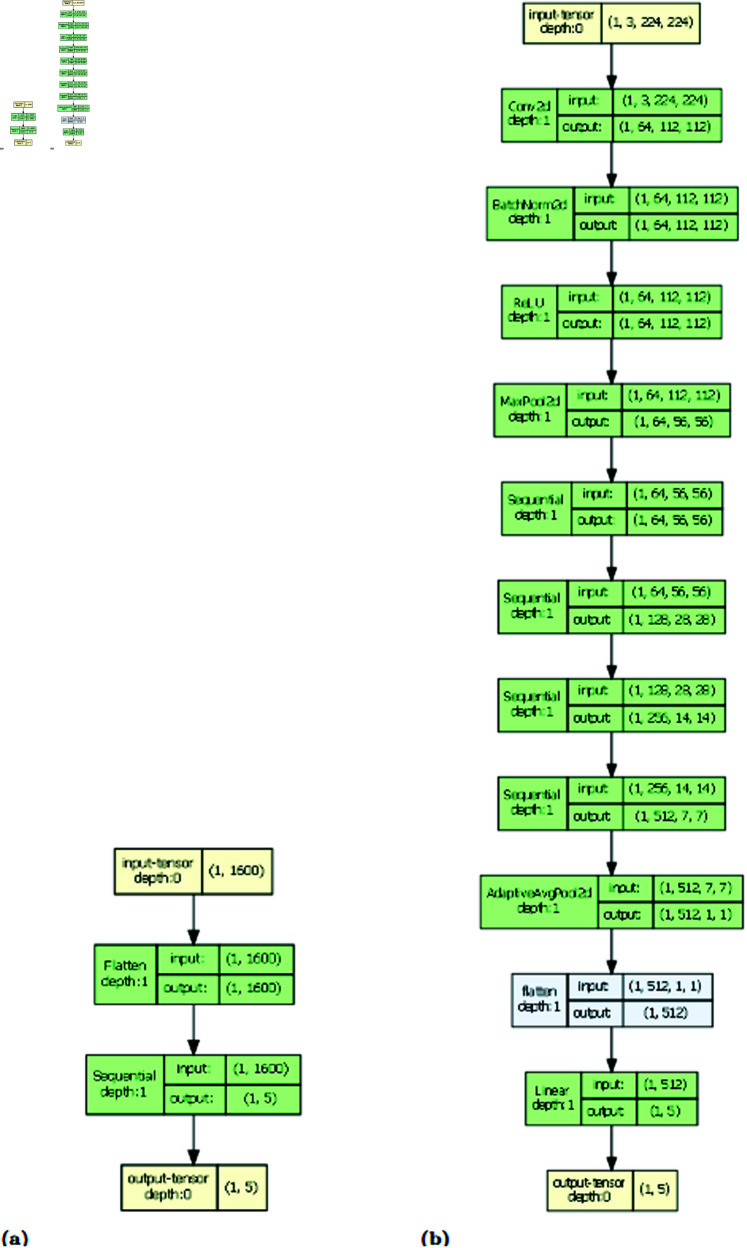
Conceptual diagrams detailing the architecture of TDANet (A) and ResNet (B). For TDANet the input is a 40×40 matrix of persistence landscape values for a stem cell colony at a particular timepoint, flattened into a one-dimensional vector. Output is a vector of 5 entries detailing the likelihoods the input colony was a protocol treatment. For ResNet, we changed the standard ResNet architecture so that its output layer was replaced with the same output layer for TDANet. Graph model made using torchview package [22].

We used a feedforward neural network, which we call *TDANet*, to interpret the colonies’ homology data. The network consists of three dense 20-neuron hidden layers with the ReLu activation, and a 5-neuron output layer with the Softmax activation function. The output consists of a “vector of probabilities”, with each index corresponding to a treatment type, and whose entry represents the likelihood the machine has that the input colony is of that type. We used categorical cross-entropy as our loss function, which is given by


$$L(x,y)=-\sum_{i}y_{i}\ln 𝒩(x{)}_{i},$$

where 𝒩() represents the output of the network and (*x*,*y*) is an input-output pair. Our code for the models, as well as processing the coordinate data into barcodes and then persistence landscapes, can be found on Github:

https://github.com/aruysdeperez/TDANet.git. See Fig 14A for a conceptual diagram of TDANet.

### Convolutional neural network

To compare our method with more traditional image classifiers, we also trained the convolutional neural network ResNet on the images of the colonies. This model is the 18-layer pretrained version provided by PyTorch [21]. We replaced the output layer with one classifying our five treatment categories. For training this model, we froze all the parameters except for our new output layer, so only that layer would be updated. See Fig 14B for a conceptual diagram of ResNet.

We trained both of our neural networks on a timepoint by timepoint basis. That is, we fixed a timepoint and trained solely on colony data from that particular timepoint. Our justification is that the network’s performance is based on time elapsed since treatment: the network will be more successful at distinguishing colonies at later timepoints, as these will be more differentiated. Thus, in training timepoint by timepoint we could establish a graded measure of the network’s accuracy based on the differentiation of the cells.

### Training and randomized labels

For both neural networks, in all cases except one, we divided the timepoint data such that 70% would be used for training, and the rest for validation. (The exception was for the work in the “Networks show similar performance...” subsection of Results, where we had an 80-20 split between training and validation.) The role of the validation data goes beyond testing. Each epoch, the neural network adjusts its parameters based on predictions made on the training data. The network then makes predictions on the validation data, but from these results makes no adjustments. The purpose is to prevent overfitting: if the accuracy on the validation data begins to decline, the network is no longer learning about the classes in general, just their selections in the training data. Thus, we save the parameters of the model at each timestep, and after several consecutive decreases in validation accuracy we stop training, as the decline suggests we are now overtraining. We take as output the version whose parameters give the highest validation accuracy.

To measure the significance of the biological classifications, we also performed training on the colonies with randomized labels. That is, instead of giving the correct treatment label to a colony, we used a label that was randomly chosen from one of the five possible. See Table 1 for the randomized distribution of labels. The training of the randomized labels also underwent a 70-30 split.

**Table 1:**
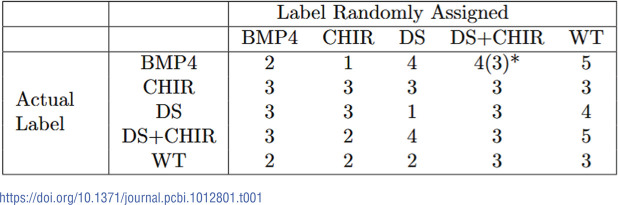
Confusion matrix for the randomized label set used to control for the performance of TDANet and ResNet. The (*i*,*j*)th entry represents the number of colonies whose actual label was *i*, but were assigned label *j*. *There was one BMP4 colony randomly labeled as DS+CHIR, for which we had colony images but lacked the cell coordinate data. Thus, while we could use this colony for training ResNet, it was not available for use by TDANet.

### Hardware and network training specifications

We constructed our nets using the PyTorch library [21]. We used cross-entropy loss for our loss function for both networks, although the nets used different optimizers: Stochastic Gradient Descent (SGD) for ResNet and Adam for TDANet. The models were trained up to a maximum of 50 epochs (in the case of ResNet) or 200 epochs (in the case of TDANet). The TDANet model has a total of 16,965 parameters, all trainable. While the ResNet model has 11,179,077 potential parameters, because we froze all but the final layer during training, there were only 2,565 trainable parameters.

We trained both neural networks on a laptop with an AMD Ryzen 7 5800H CPU (running at base clock speed 3.20 GHz) and 16.0 GB of RAM. The typical training session took around 5 seconds for a TDANet model and around a minute for a ResNet model.

## References

[1] Warmflash A, Sorre B, Etoc F, Siggia ED, Brivanlou AH. A method to recapitulate early embryonic spatial patterning in human embryonic stem cells. Nat Methods. 2014;11(8):847–54. doi: 10.1038/nmeth.3016 24973948 PMC4341966

[2] Hookway TA, Butts JC, Lee E, Tang H, McDevitt TC. Aggregate formation and suspension culture of human pluripotent stem cells and differentiated progeny. Methods. 2016;101:11–20. doi: 10.1016/j.ymeth.2015.11.027 26658353

[3] Godinez WJ, Hossain I, Lazic SE, Davies JW, Zhang X. A multi-scale convolutional neural network for phenotyping high-content cellular images. Bioinformatics. 2017;33(13):2010–9. doi: 10.1093/bioinformatics/btx069 28203779

[4] Nitta N, Sugimura T, Isozaki A, Mikami H, Hiraki K, Sakuma S, et al. Intelligent image-activated cell sorting. Cell. 2018;175(1):266-276.e13. doi: 10.1016/j.cell.2018.08.028 30166209

[5] Liu W, Li C, Rahaman MM, Jiang T, Sun H, Wu X, et al. Is the aspect ratio of cells important in deep learning? A robust comparison of deep learning methods for multi-scale cytopathology cell image classification: From convolutional neural networks to visual transformers. Comput Biol Med. 2022;141:105026. doi: 10.1016/j.compbiomed.2021.105026 34801245

[6] Buggenthin F, Buettner F, Hoppe PS, Endele M, Kroiss M, Strasser M, et al. Prospective identification of hematopoietic lineage choice by deep learning. Nat Methods. 2017;14(4):403–6. doi: 10.1038/nmeth.4182 28218899 PMC5376497

[7] Kim JM, Moon S-H, Lee SG, Cho YJ, Hong KS, Lee JH, et al. Assessment of differentiation aspects by the morphological classification of embryoid bodies derived from human embryonic stem cells. Stem Cells Dev. 2011;20(11):1925–35. doi: 10.1089/scd.2010.0476 21388292

[8] Kovacev-Nikolic V, Bubenik P, Nikolić D, Heo G. Using persistent homology and dynamical distances to analyze protein binding. Stat Appl Genet Mol Biol. 2016;15(1):19–38. doi: 10.1515/sagmb-2015-0057 26812805

[9] Bhaskar D, Zhang WY, Wong IY. Topological data analysis of collective and individual epithelial cells using persistent homology of loops. Soft Matter. 2021;17(17):4653–64. doi: 10.1039/d1sm00072a 33949592 PMC8276269

[10] Joy DA, Libby ARG, McDevitt TC. Deep neural net tracking of human pluripotent stem cells reveals intrinsic behaviors directing morphogenesis. Stem Cell Reports. 2021;16(5):1317–30. doi: 10.1016/j.stemcr.2021.04.008 33979602 PMC8185472

[11] Bargaje R, Trachana K, Shelton MN, McGinnis CS, Zhou JX, Chadick C, et al. Cell population structure prior to bifurcation predicts efficiency of directed differentiation in human induced pluripotent cells. Proc Natl Acad Sci U S A. 2017;114(9):2271–6. doi: 10.1073/pnas.1621412114 28167799 PMC5338498

[12] Yanagihara K, Liu Y, Kanie K, Takayama K, Kokunugi M, Hirata M, et al. Prediction of differentiation tendency toward hepatocytes from gene expression in undifferentiated human pluripotent stem cells. Stem Cells Dev. 2016;25(24):1884–97. doi: 10.1089/scd.2016.0099 27733097 PMC5165660

[13] Zhou B, Khosla A, Lapedriza A, Oliva A, Torralba A. Learning deep features for discriminative localization. In: Proceedings of the IEEE Conference on Computer Vision and Pattern Recognition. 2016. p. 2921–9.

[14] Edelsbrunner H, Letscher D, Zomorodian A. Topological persistence and simplification. In: Proceedings 41st Annual Symposium on Foundations of Computer Science. 2000. p. 454–63.

[15] Bauer U. Ripser: efficient computation of vietoris–rips persistence barcodes. J Appl Comput Topol. 2021;5(3):391–423. doi: 10.1007/s41468-021-00071-5

[16] Niehrs C. The complex world of WNT receptor signalling. Nat Rev Mol Cell Biol. 2012;13(12):767–79. doi: 10.1038/nrm3470 23151663

[17] Wang RN, Green J, Wang Z, Deng Y, Qiao M, Peabody M, et al. Bone Morphogenetic Protein (BMP) signaling in development and human diseases. Genes Dis. 2014;1(1):87–105. doi: 10.1016/j.gendis.2014.07.005 25401122 PMC4232216

[18] Papanayotou C, Collignon J. Activin/Nodal signalling before implantation: setting the stage for embryo patterning. Philos Trans R Soc Lond B Biol Sci. 2014;369(1657):20130539. doi: 10.1098/rstb.2013.0539 25349448 PMC4216462

[19] Aragón E, Wang Q, Zou Y, Morgani SM, Ruiz L, Kaczmarska Z, et al. Structural basis for distinct roles of SMAD2 and SMAD3 in FOXH1 pioneer-directed TGF-β signaling. Genes Dev. 2019;33(21–22):1506–24. doi: 10.1101/gad.330837.119 31582430 PMC6824466

[20] Bubenik P, Dłotko P. A persistence landscapes toolbox for topological statistics. J Symbol Comput. 2017;78:91–114. doi: 10.1016/j.jsc.2016.03.009

[21] Paszke A, Gross S, Massa F, Lerer A, Bradbury J, Chanan G, et al. Pytorch: an imperative style, high-performance deep learning library. Adv Neural Inf Process Syst. 2019;32.

[22] Yandell H. mert-kurttutan/torchview; 2024. 10.5281/zenodo.14004786

[23] Omeiza D, Speakman S, Cintas C, Weldermariam K. Smooth grad-cam: an enhanced inference level visualization technique for deep convolutional neural network models. arXiv preprint 2019. https://arxiv.org/abs/1908.01224

